# Myocardial infarction activates the 9p21.3 orthologous locus expression, but its absence does not alter cardiac pathophysiology in ischemia

**DOI:** 10.14814/phy2.70344

**Published:** 2025-05-22

**Authors:** Sanna Kettunen, Anna Slita, Tuisku Suoranta, Iida Räty, Svetlana Laidinen, Elias Ylä‐Herttuala, Anna‐Kaisa Ruotsalainen, Seppo Ylä‐Herttuala

**Affiliations:** ^1^ A.I. Virtanen Institute University of Eastern Finland Kuopio Finland; ^2^ Clinical Radiology Unit Kuopio University Hospital Kuopio Finland; ^3^ Heart Center and Gene Therapy Unit Kuopio University Hospital Kuopio Finland

**Keywords:** 9p21.3, Ak148321, ANRIL, LAD ligation, myocardial infarction

## Abstract

Genetic variation in the 9p21.3 chromosomal region has one of the strongest associations known for coronary artery disease (CAD) that often leads to myocardial infarction (MI). This risk locus encodes a long noncoding RNA, ANRIL, which has been suggested to regulate the neighboring cyclin‐dependent kinase inhibitors 2A and B (*Cdkn2A/B*), the key regulators of cell proliferation. In this study, we aimed to clarify the role of the 9p21.3 risk locus in acute and chronic myocardial ischemia in mice. Mice carrying a deletion equivalent to the human CAD risk interval (Chr4^Δ70kb/Δ70kb^) and wild type mice were exposed to MI and followed until 5 days or 4 weeks. In the wild type mice, expression of a lncRNA, Ak148321, was increased after MI, and Cdkn2a was upregulated in chronic ischemia. Chr4^Δ70kb/Δ70kb^ downregulated both *Cdkn2a/b*, but this did not affect the survival or cardiac pathology after MI. These results suggest that the 9p21.3 locus is activated in response to cardiac ischemia. However, deficiency in the risk locus does not play a role in the cardiac pathophysiology in mice, supporting the studies suggesting the risk locus being more involved in the development of CAD, rather than the subsequent MI.

## 1 INTRODUCTION

Cardiovascular diseases and subsequent myocardial infarction (MI) are the leading cause of death worldwide (Vaduganathan et al., 2022). MI is typically caused by atherosclerotic coronary artery disease (CAD), a condition where oxidized low‐density lipoproteins, cholesterol, and inflammatory cell accumulation into the arterial wall leads to the formation of a complex plaque (Lusis, 2000; Ylä‐Herttuala et al., 1989). Plaques obstruct coronary arteries, and plaque erosion or rupture can lead to arterial occlusion and myocardial ischemia. Genome‐wide association studies (GWAS) have revealed a locus in the 9p21.3 chromosomal region being a hotspot for CAD‐associated genetic variation (Helgadottir et al., 2007; McPherson et al., 2007; Samani et al., 2007; The Wellcome Trust Case Control Consortium, 2007). This risk locus encodes a large antisense noncoding RNA ANRIL which has been suggested to play a role in epigenetic regulation, miRNAs sponging, and modulation of inflammatory responses (Kettunen et al., 2023; Kotake et al., 2011; Zeng et al., 2019; Zhou et al., 2016). However, the role of 9p21.3 and ANRIL in MI has been disputed. While variation in the 9p21.3 locus and ANRIL expression have been associated with the risk of MI in some studies, other studies focused on patients with established CAD have revealed no significant link between the 9p21.3 genotype and CAD outcomes, including MI (Cheng et al., 2017; Haslacher et al., 2016; Horne et al., 2008; Patel et al., 2014, 2019; Tibaut et al., 2022). In hypertensive patients, the 9p21.3 locus variation was independently associated with stroke (Wahlstrand et al., 2009).

Expression of lncRNAs, including ANRIL, has been shown to be altered after MI, and ANRIL has been suggested to play a role in the regulation of angiogenesis and apoptosis after myocardial ischemia (Huang, Zhao, et al., 2020; Jiao et al., 2023; Rodríguez‐Esparragón et al., 2023; Vausort et al., 2014; Yang et al., 2019). Moreover, ANRIL has both linear (lin) and circular (circ) splicing isoforms, and several studies have demonstrated them having different effects, as the linear isoforms seem to be associated with an elevated risk for CVDs, while circANRIL has been associated with protection towards both CAD and MI (Burd et al., 2010; Holdt et al., 2016; Razeghian‐Jahromi et al., 2022; Rodríguez‐Esparragón et al., 2023). ANRIL has been shown to regulate its neighboring Cyclin Dependent Kinase Inhibitor genes, *CDKN2A* and ‐*B*, but little is known about their role in myocardial ischemia (Cho et al., 2019; Kotake et al., 2011; Yap et al., 2010). Both *CDKN2A* and ‐*B* are expressed in human carotid plaques, and one study reported lower levels of *CDKN2B* in the peripheral blood of MI patients and carriers of a 9p21.3 risk allele (Holdt et al., 2011; Zivotić et al., 2019). Another study found lower *CDKN2B* expression in carotid arteries of CAD patients in comparison to healthy subjects (Cho et al., 2019). Nevertheless, *CDKN2A* and ‐*B* are important regulators of cell proliferation, a process necessary for the repair and revascularization after tissue damage, like ischemia.

A transgenic mouse model targeting a similarly located sequence to human CAD risk interval has been generated, and in a proatherogenic background, these Chr4^Δ70kb/Δ70kb^ mice develop increased advanced atherosclerosis due to increased smooth muscle cell proliferation and macrophage pro‐inflammatory activity when subjected to a high fat diet (Kettunen et al., 2023; Kojima et al., 2020; Visel et al., 2010). In the present study, we studied the implications of this risk locus orthologue knockout on the survival, cardiac function, and recovery of the myocardium after both acute and chronic MI in mice. We subjected the Chr4^Δ70kb/Δ70kb^ and wild type (WT) mice to myocardial ischemia by a permanent ligation of the left ascending coronary artery (LAD) and investigated their cardiac function, as well as the severity of the cardiac injury and the postischemic myocardial remodeling. Our data demonstrate that the 9p21.3 murine orthologous locus is activated in myocardial ischemia, and it regulates the *Cdkn2a/b* expression in response to myocardial ischemia, but without effects on the survival or the severity of the cardiac pathology.

## 2 METHODS

### 2.1 Myocardial infarction model

**FIGURE 1:**
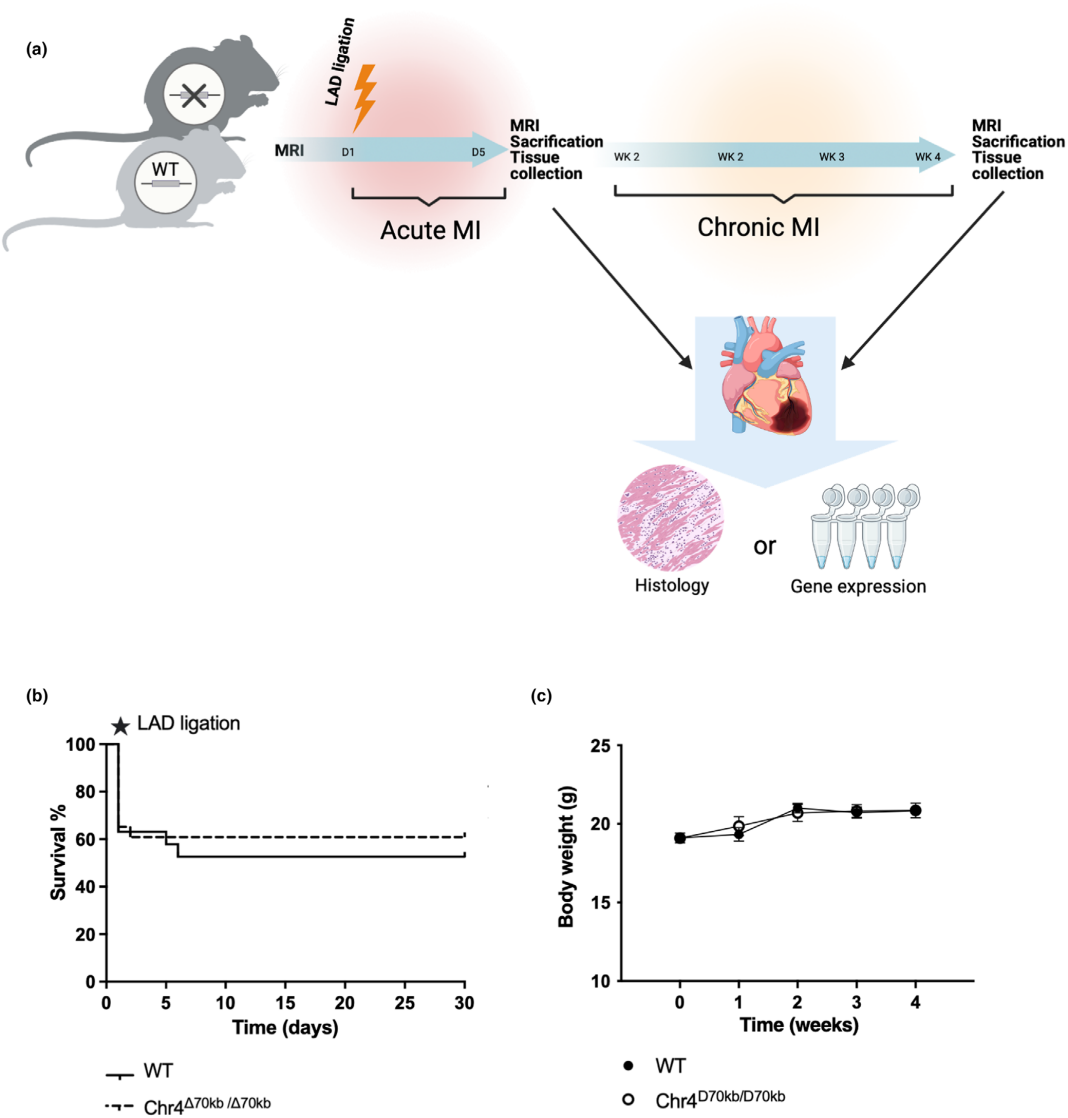
(a) Study protocol. Chr4^D70/D70^ (*n* = 20) and wild type (WT) (*n* = 15) C57Bl/6JOlaHsd female mice were subjected to LAD ligation to induce myocardial infarction (MI) and followed until 5 days or 4 weeks (*n* = 24 + 19) post operation, after which they were euthanized, and their tissues were collected. (b) 4 weeks survival of Chr4^D70/D70^ (*n* = 24) and WT (*n* = 19) mice after MI. (c) Weight development of Chr4^D70/D70^ (*n* = 7–22) and WT mice (*n* = 6–19) after MI.

To study the role of the murine equivalent of human Chr9p21.3 CAD risk locus and ANRIL in the context of both acute and chronic myocardial infarction, Chr4^Δ70kb/Δ70kb^ mice from MMRRC (RRID:MMRRC_032091‐UCD) mice were first crossbred into a C57Bl/6JOlaHsd wild type background (originating from Inotiv, West Lafayette, IN, USA). Myocardial ischemia was surgically induced in 10‐week‐old female Chr4^Δ70kb/Δ70kb^ and wild type (WT) mice by permanent ligation of the left anterior descending coronary artery (LAD), performed by utilizing the rapid pop‐out method (Gao et al., 2010). Female mice are known to be more susceptible to atherosclerotic CVD, the most common cause of MI in humans (Christ et al., 2024; Robinet et al., 2018). Moreover, in our previous study with Chr4^Δ70kb/Δ70kb^ female mice, we found them developing an increased atherosclerosis but also an increased macrophage pro‐inflammatory activity when subjected to pro‐atherogenic background and diet (Kettunen et al., 2023). During the LAD‐ligation operation, mice were under isoflurane inhalation anesthesia (4% induction, 2% maintenance) and received analgesia (10 mg/kg carprofen once a day and 0.1 mg/kg buprenorphine twice a day s.c) prior to the operation and for the following 2 days. Chr4^Δ70kb/Δ70kb^ and WT mice were then followed until 5 days (*n* = 20 + 15) or 4 weeks (*n* = 24 + 19) post‐ligation (Figure 1a). After that, the mice were sacrificed with CO_2_ and their hearts were collected for either histology or gene‐expression analyses. LAD ligation was considered successful when the MI area was >5% as determined by cardiac histology. Mice with <5% MI area were excluded from further analysis. The MI area could not be determined from the hearts that were collected for gene expression analysis. The exact number of the animals for each analysis is provided in the methods and in each figure legend. Survival analysis and body weight were recorded from the 4‐week study group. Beyond the survival and weight data, prematurely died animals were not included in further analysis. Mice were housed in the Animal Centre of the University of Eastern Finland and received food (Teklad Global 2016, 16% Protein Rodent Diet: 12% of calories from fat and 0% cholesterol) and water ad libitum.

### 2.2 Histology

Heart samples for histology were collected from the euthanized mice and fixed in 4% formaldehyde in phosphate buffered saline (PBS, pH 7.4) overnight, after which they were processed and embedded into paraffin. The number of Chr4^Δ70kb/Δ70kb^ mice for each histological analysis at both 5 days and 4‐weekss time points was 5, and for WT mice it was 4. Whole hearts were first cut into 4 μm thick cross‐sections collected at 40 μm intervals. Each analysis was then performed for 4–10 individual cross‐sections collected at different levels of the heart from apex to base, and their average was calculated. All histological sections were imaged with an ECLIPSE Ni‐E microscope (Nikon instruments Inc., Tokyo, Japan) and analyzed with Fiji software (Schindelin et al., 2012).

For MI area analysis, sections were stained with hematoxylin‐eosin (H&E) or Masson's Trichrome. At 5‐days' time point, the MI area was recorded from H&E‐stained Chr4^Δ70kb/Δ70kb^ and WT heart sections by drawing a region‐of‐interest (ROI) around the apoptotic cardiomyocytes and infiltrated immune cells and measuring the area in relation to the whole LV myocardium. From the stabilized and already fibrotic 4‐week post‐operation hearts, the MI scar area of the Chr4^Δ70kb/Δ70kb^ and WT animals was analyzed from Masson trichrome (Sigma‐Aldrich) stained cross‐sections by using Fiji software color threshold tool recognizing the fibrotic scar (Schindelin et al., 2012). Mice with <5% MI area were excluded from further analysis.

Acerage cardiomyocyte sizes were analyzed from H&E‐stained high magnification cross‐sections of myocardium by drawing a ROIs around 5–10 randomly selected cardiomyocytes per mouse and calculating their area. For immune cell infiltration, cardiac cross sections were immunostained with MAC3 primary antibody (553322, BD Pharmingen™, Becton, Dickinson and Company, Franklin Lakes, NJ, USA) for macrophages and CD4 primary antibody (D7D2Z, Cell Signalling Technology, Danvers, MA, USA) for T‐lymphocytes, and with appropriate secondary antibodies (BA‐1000 Goat‐anti‐rabbit IgG for CD4 and BA‐4001 Rabbit‐anti‐rat IgG for MAC3, by VectorLabs Newark, CA, USA). MAC3 and CD4 positive areas were then analyzed by using the Fiji color threshold tool recognizing the DAB positive staining area and represented as a percentage (%) of the whole LV myocardium. Edema of the LV myocardium, excluding the scar, was analyzed from the hematoxylin‐eosin‐stained sections by measuring the area of empty space between the cardiomyocytes and presented as a percentage of the whole LV myocardium. For ischemia induced revascularization and capillaries, cardiac cross‐sections were immunostained with Podocalyxin primary antibody (AF1556, R&D systems, Bio‐Techne, Minneapolis, MN, USA). The capillary density in the LV myocardium was analyzed by detecting Podocalyxin positive staining area.

### 2.3 Imaging and cardiac function

Hearts of randomly selected WT (*n* = 14) and Chr4^Δ70kb/Δ70kb^ (*n* = 17) mice were imaged before and after 5 days (*n* = 4 + 5) or 4 weeks (*n* = 4 + 5) of MI in vivo with a horizontal 7 T magnet (Bruker BioSpin, Ettlingen, Germany, with ParaVision 6.01) or a 9.4 T magnet (Varian Inc. Palo Alto, California, USA) controlled by a Bruker console (Bruker GmbH, Ettlingen, Germany, with ParaVision 5.1) (Table S1). Prior to imaging, mice were anesthetized with 4% isoflurane in a 70% N_2_ and 30% O_2_ gas mixture, and the anesthesia was maintained with isoflurane levels of 1%–2.5% during the imaging. The body temperature of the mice was sustained with a warm water pad. Mouse hearts, including the full cardiac cycle, were imaged by applying fast imaging with balanced steady‐state precession (FISP) or dynamic FLASH readout sequence. The number of frames was 10–11 depending on the heart rate, and 10–14 single slices from the short axis view were acquired. Electrocardiogram (ECG) was measured either from the forepaws of the mice using needle electrodes, or with IntraGate (Bruker Biospin, Ettlingen, Germany.) Respiration of the animals was monitored by a pneumatic pillow placed under the mice. Both signals were registered using Model 1025 monitoring (Small Animal Instruments Inc., Stony Brook, New York, USA).

Cardiac function of the mice was analyzed from the MRI by using MATLAB (MathWorks Inc., Natick, CA, USA) with Aedes Software (http://aedes.uef.fi). Axial cine images from the center of the heart were selected, and the surface area of the left ventricle in both diastole and systole was determined. Ejection fraction (EF), stroke volume (SV), end‐diastolic volume (EDV) and end‐systolic volume (ESV) were calculated based on these values.

### 2.4 Gene expression analysis

For the cardiac gene expression analysis, mouse hearts were harvested from the euthanized mice and immediately placed into liquid N2. In addition to the MI mice, hearts of five nonoperated WT mice were also collected to demonstrate the expression of the genes of interest in a healthy mouse myocardium. The RNA was extracted with phenol (15596026, TRI Reagent™ Solution, Invitrogen™, Thermo Fisher Scientific, Waltham, MA, USA) and treated with the DNA‐free™ DNA Removal Kit (AM1906, Invitrogen™, Thermo Fisher Scientific, Waltham, MA, USA). The mRNA expression of the CAD risk locus exons and circular transcripts was measured from RNA samples by using One‐step Droplet Digital PCR (Bio‐Rad laboratories, Hercules, CA, USA). Briefly, 50 ng of RNA was mixed with the ingredients of the One‐Step RT‐ddPCR Advanced Kit for Probes (1864022, Bio‐Rad laboratories, Hercules, CA, USA) according to the manufacturer's protocol. The droplets were generated with the QX200™ Automated Droplet Generator using the Droplet Generator Oil for Probes (1864110, Bio‐Rad laboratories, Hercules, CA, USA). The reverse transcription and annealing stage were performed with the C1000 Thermal Cycler (Bio‐Rad) and the temperatures were determined during several optimization runs. After 3–18 h stabilization at 8°C, results were read on the QX200 Droplet Reader (Bio‐Rad laboratories, Hercules, CA, USA) and analyzed using QX Manager Software. The data was normalized to an endogenous control *Rplp0* (Table S2), and values were compared between Chr4^Δ70kb/Δ70kb^ and WT mice.

For the expression of genes regulating cell cycle and cardiac remodeling, the scar and healthy tissues were first separated from the selected Chr4^Δ70kb/Δ70kb^ and WT mice at the 4 weeks' time point. The RNA was then extracted and treated as described above and reverse transcribed into cDNA (K1622, RevertAid First Strand cDNA Synthesis Kit, Invitrogen™, Thermo Fisher Scientific, Waltham, MA, USA) by using random hexamer primers (SO142, Thermo Fisher Scientific, Waltham, MA, USA). MRNA expression of the target genes was measured by using quantitative Polymerase Chain reaction (qPCR) (StepOnePlus™ Real‐Time PCR System, Applied Biosystems, Thermo Fisher Scientific, Waltham, MA, USA), with TaqMan™ Universal PCR Master Mix (4304437, Applied Biosystems, Thermo Fisher Scientific, Waltham, MA, USA) and TaqMan based assays from Integrated DNA Technologies (Coralville, IA, USA) and Thermo Fisher Scientific (Waltham, MA, USA) (Table S2). The measured mRNA levels were normalized to endogenous control *Hprt1*, and the relative gene expression levels were analyzed by using the 2–∆∆Ct method.

### 2.5 Statistics

All numerical results in the text and graphs are represented as a mean ± standard deviation. All statistical analyses were performed with GraphPad Prism (Version 9.3.1, GraphPad Software LLC, Boston, MA, USA) analysis tools. Survival curves were compared by using the Log‐rank (Mantel‐Cox) test. The difference in means between two groups was measured by using the *t*‐test. The difference in means between more than two groups was measured by using ANOVA with Tukey's multiple comparisons test, and when comparing both genotypes and matching samples from the same mouse (intact and infarcted tissue), Fisher's LSD multiple comparisons test was used. Correlation between two variables was tested with the Pearson *r*‐test. Statistical tests used are mentioned in each figure legend. In all statistical tests, differences were considered statistically significant when *p* or adjusted *p* < 0.05.

## 3 RESULTS

### 3.1 The expression of Ak148321 and *Cdkn2a/b* is upregulated in the infarcted myocardium in wild type but not in Chr4^Δ70kb^ ^/Δ70kb^ mice

Survival rates of Chr4^Δ70kb/Δ70kb^ (60.87%) and wild type mice (52.63%) did not differ during the 4 weeks follow‐up after the MI (Figure 1b). In both groups, most of the deaths occurred during or right after the operation. There was no difference in body weight of the animals during the follow‐up either (Figure 1c).

**FIGURE 2:**
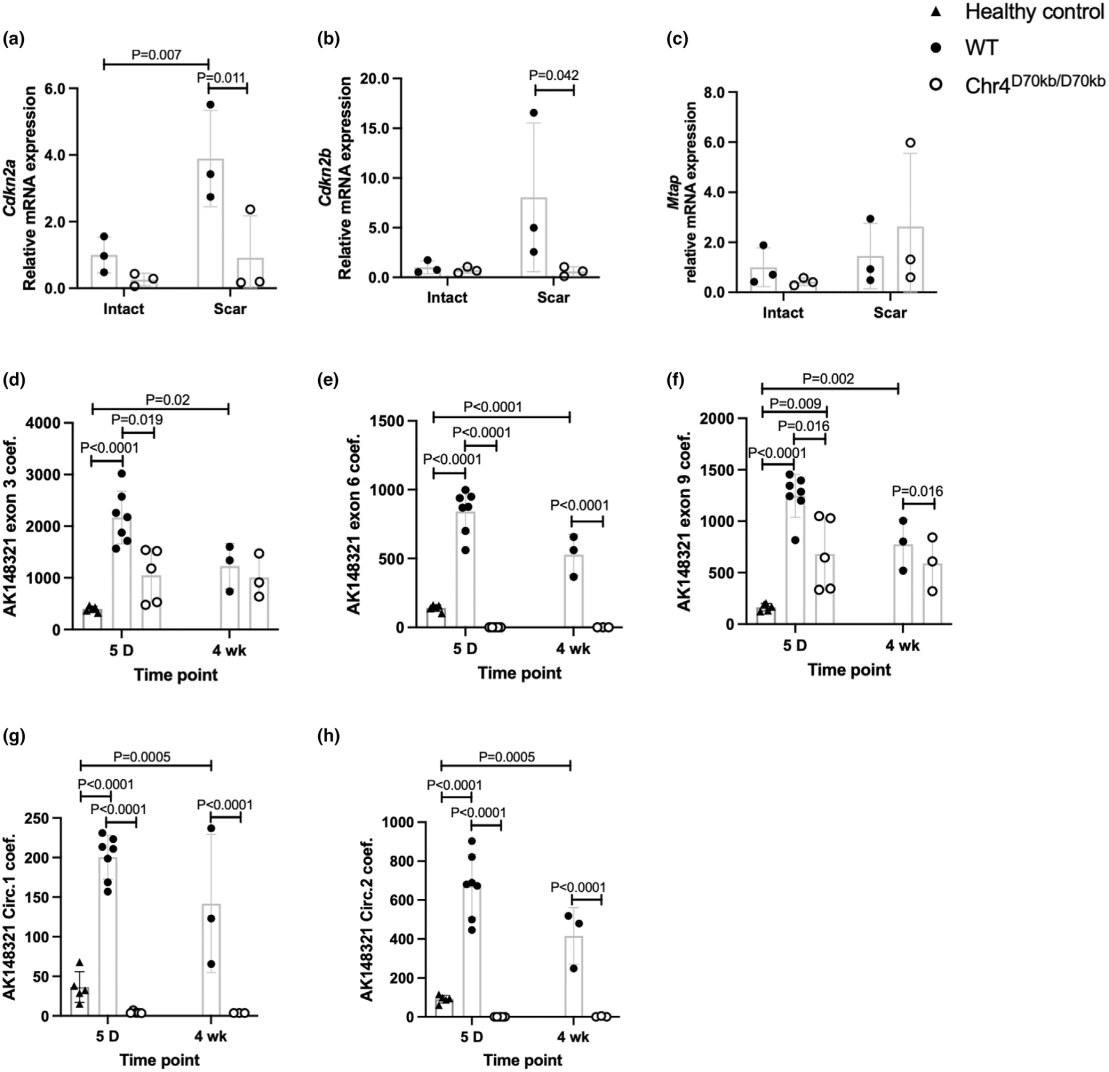
In chronic, 4 weeks myocardial infarction, cardiac expression of *Cdkn2a* was increased in the scar area of wild type mice, but not in Chr4^d70/d70^ mice. The AK148321 expression is promoted by myocardial infarction. Cardiac expression of (a) *Cdkn2a*, (b) *Cdkn2b*, (c) *Mtap* in Chr4^d70/d70^ (*n* = 3) and WT (*n* = 3) mice's intact and scarred LV myocardium in chronic, 4 weeks' time point MI. (d) Relative mRNA expression of Ak148321 exon 3, (e) exon 6, (f) exon 9 and its (g) circular isoform 1 and (h) circular isoform 2 in WT (*n* = 7) and Chr4^d70/d70^ (*n* = 5) mice at day 5 and after 4 weeks (*n* = 3 + 3) of myocardial infarction, as well as in healthy WT mice with no MI (*n* = 5). mRNA expression for the protein coding genes *Cdkn2a/b* and *Mtap* was measured with qPCR and normalized to an endogenous control Hprt1. mRNA expression of Ak148321 was measured with ddPCR and the data was normalized to an endogenous control *Rplp0*. Statistical analysis for graphs (a)–(c) was performed with Two‐tailed ANOVA with Fisher's LSD multiple comparisons test and with Tukey's multiple comparisons test for the rest. Difference between groups was considered statistically significant when *p* < 0.05.

To investigate the potential effects of the risk locus on cardiac repair after MI, we dissected and separated the scar and intact myocardium from a couple of chronically infarcted Chr4^Δ70kb/Δ70kb^ and WT mouse hearts (*n* = 3 + 3) and measured the expression levels of genes important for cell proliferation and cardiac remodeling, neighboring the CAD risk locus. There were no differences in the expression of cell cycle regulators *Cdkn2a* (Figure 2a), *Cdkn2b* (Figure 2b), or *Mtap* (Figure 2c) between the Chr4^Δ70kb/Δ70kb^ and WT mice hearts in the intact tissue, but in the scar area, the WT mice showed significantly increased mRNA expression of *Cdkn2a* (*p* = 0.007) and *‐b* (*p* = 0.042) in comparison to Chr4^Δ70kb/Δ70kb^ mice (Figure 2a,b). The expression of *Cdkn2a* was significantly increased in the WT mice infarction area in comparison to their intact myocardium (*p* = 0.011), while in the Chr4^Δ70kb/Δ70kb^ mice, the expression remained at a low level. *Cdkn2b* showed a similar trend, yet without statistical significance.

To investigate the risk locus expression in the acute and chronic MI, we measured the cardiac RNA expression of three Ak148321 exons and two circular isoforms in 5 days and 4 weeks post MI mice and in healthy WT mice. All three Ak148321 exons, 3 (*p* = 0.019), 6 (*p* < 0.0001) and 9 (*p* = 0.016), and the circular isoforms (Circ1 *p* < 0.0001); Circ2 *p* < 0.0001 were downregulated in Chr4^Δ70kb/Δ70kb^ mice in the acute 5‐day time point when compared to WT (Figure 2d–h). However, at the 4‐week time point, exon 3 was not downregulated in Chr4^Δ70kb/Δ70kb^ mice (Figure 2d). Interestingly, the WT expression of all the Ak148321 exons (Exon 3; *p* < 0.0001; exon 6 *p* < 0.0001; exon 9 *p* < 0.0001) and the circular isoforms (Circ1 *p* < 0.0001; Circ2 < 0.0001) were significantly higher after 5 days of infarction than in an non‐MI healthy WT hearts (Figure 2d,h). Their expression decreased in 4 weeks in MI WT hearts, but remained still significantly higher than in healthy WT mice (Exon 3; *p* = 0.02; exon 6 *p* < 0.0001; exon 9 *p* = 0.002); (Circ1 *p* = 0.0005; Circ2 *p* = 0.0005).

### 3.2 Chr4^Δ70kb^ ^/Δ70kb^ does not affect the severity of myocardial infarction in mice

**FIGURE 3:**
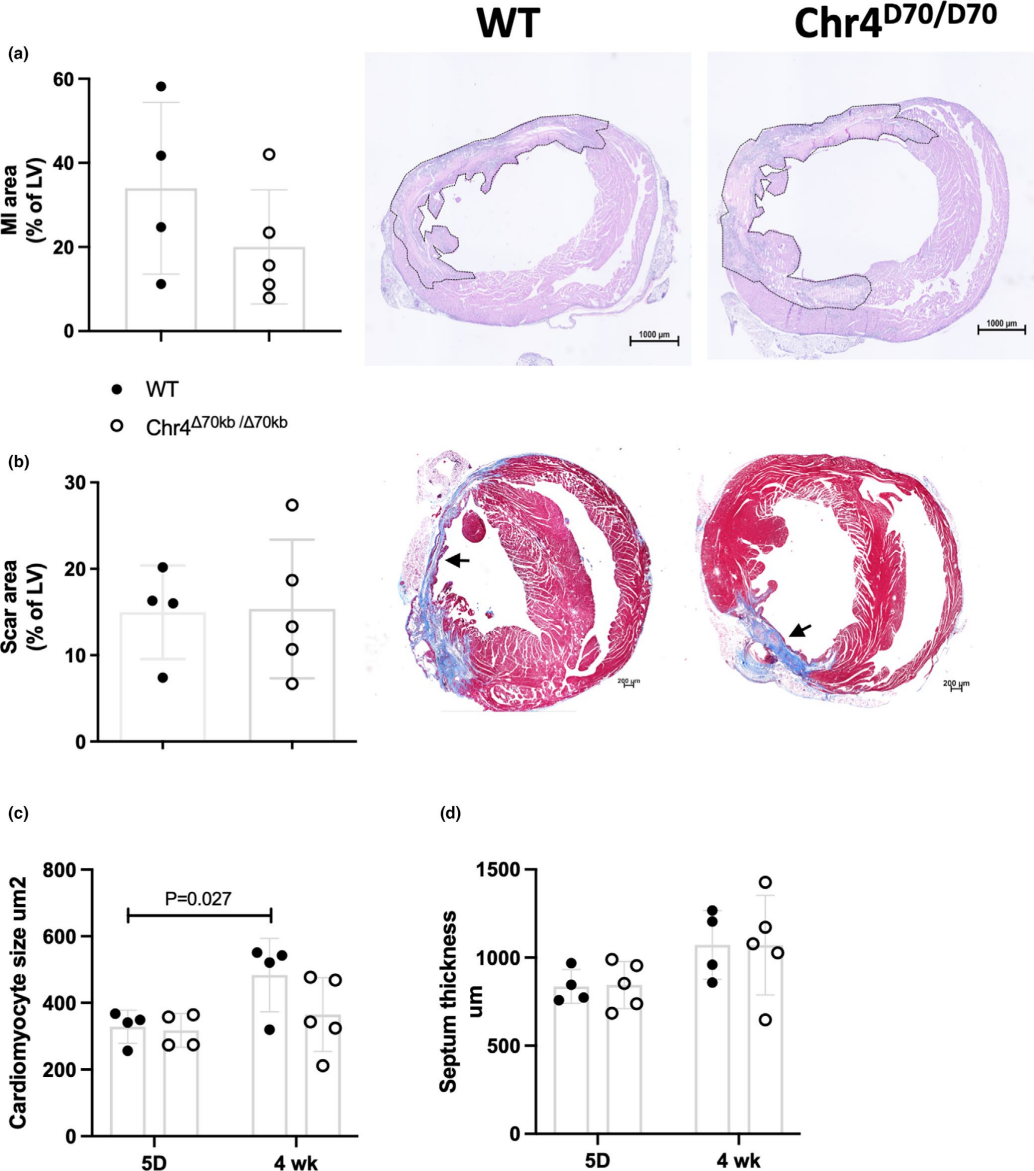
Chr4^D70/D70^ did not affect the relative infarction area or histopathology of the myocardium. (a) Infarcted area and dead cardiomyocytes in WT (*n* = 4) and Chr4^D70/D70^ (*n* = 5) mice at day 5 after the MI. MI area (indicated by dotted line) was analyzed from H&E‐stained sections and represented as a percentage area of the left ventricle myocardium. Mice with <5% MI area were excluded from the data, and further histological and imaging data. (b) Masson's Trichrome stained infarction scar area (indicated by arrowheads) of WT (*n* = 4) and Chr4^D70/D70^ (*n* = 5) mice after 4 weeks of MI. Relative scar area is represented as a percentage area of the left ventricle myocardium. Mice with <5% MI area were excluded from the data, and further histological and imaging data. (c) Average cardiomyocyte size of WT (*n* = 4) and Chr4^D70/D70^ (*n* = 4) mice at day 5 and after 4 weeks (*n* = 4 + 5) from MI. ED) Average thickness of the heart septum in WT (*n* = 4) and Chr4^D70/D70^ (*n* = 5) mice at day 5 and after 4 weeks (*n* = 4 + 5) of MI. Statistical analysis for graphs (a) and (b) was performed by using *t*‐test, and for graphs (c) and (d), two‐way mixed effects ANOVA with Tukey's multiple comparisons test was used. Differences were considered statistically significant when *p* < 0.05.

Five days after the LAD ligation, MI was still at acute phase characterized by dead or apoptotic cardiomyocytes and immune cell infiltration around the infarction site (Figure 3a), while after 4 weeks, both Chr4^Δ70kb/Δ70kb^ and WT mice had developed a narrow fibrotic infarction scar in their LV (Figure 3b). There was a high variation in the size of the MI area, ranging from below 10% to near 30%, and no difference between Chr4^Δ70kb/Δ70kb^ and WT mice was observed neither in acute nor chronic MI area (Figure 3a,b). As cardiac pathologies may result in heart failure and hypertrophy, we measured both cardiomyocyte size and the thickness of the cardiac septum from the histological sections of the Chr4^Δ70kb/Δ70kb^ and WT mice. The average size of cardiomyocytes was significantly larger in WT mice after 4 weeks of MI than in the acute 5‐days' time point (*p* = 0.027) (Figure 3c). The Chr4^Δ70kb/Δ70kb^ mice showed no difference between acute and chronic infarction due to the higher variation, and there was no difference between genotypes either. There were no differences in the septum thickness between the Chr4^Δ70kb/Δ70kb^ and WT mice, nor between the time points (Figure 3d).

**FIGURE 4:**
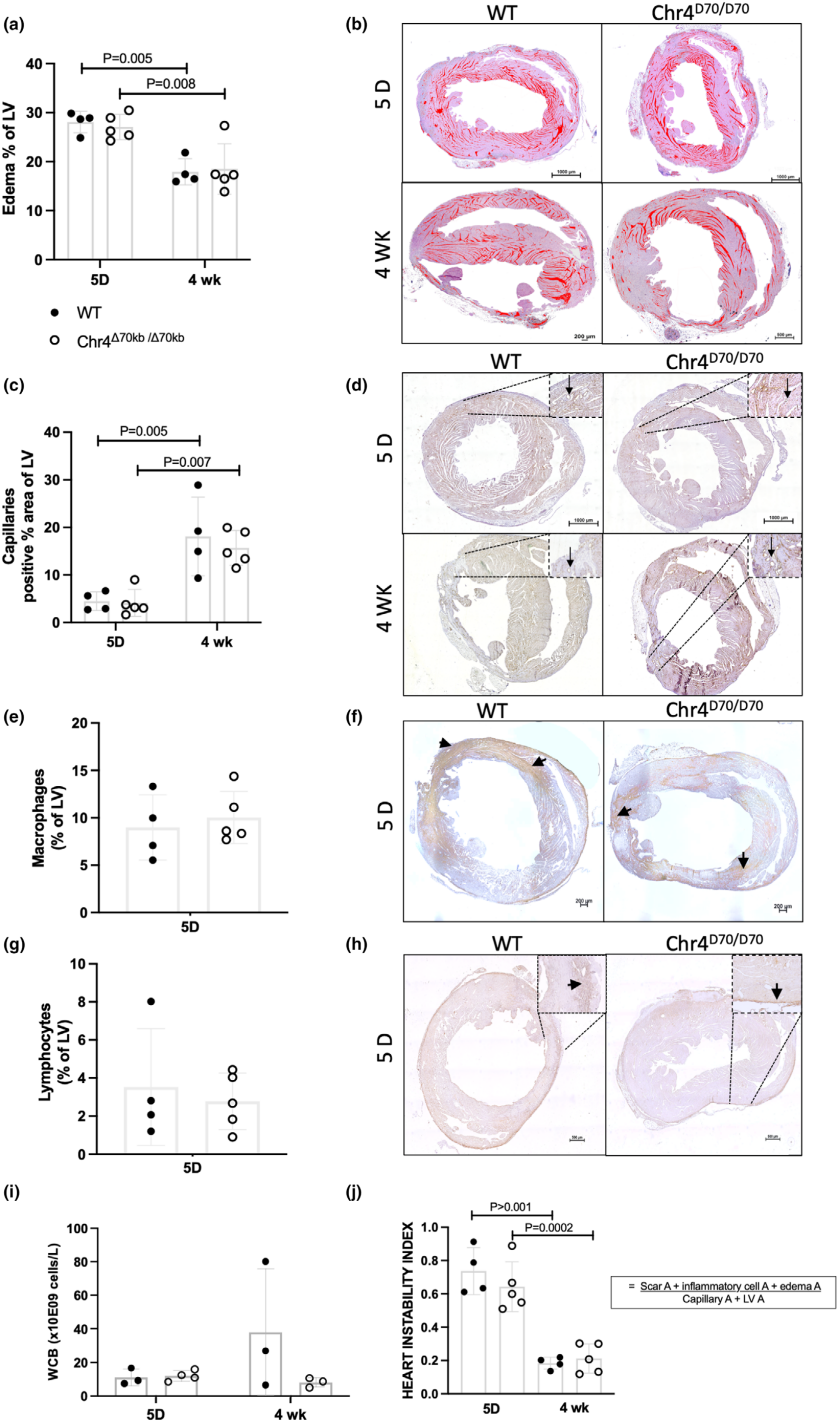
Edema of the myocardium was significantly reduced, and capillary area increased after 4 weeks of infarction, in comparison to acute condition at day 5. (a) Cardiac tissue edema of Chr4^D70/D70^ and WT hearts presented as percentage area of LV at day 5 (*n* = 4 + 5) and after 4 weeks (*n* = 4 + 5) of LAD ligation. Analyzed from H&E‐stained cardiac cross‐sections, excluding visible scar. (b) Representative figures of cardiac tissue edema, characterized as an empty space within the tissue, highlighted in red. (c) Capillary positive area in LV presented as percentage area at day 5 (*n* = 4 + 5) and after 4 weeks (*n* = 4 + 5) of MI. Analyzed from Podocalyxin primary antibody‐stained cross‐sections of the myocardium. (d) Representative figures of Podocalyxin immunostained myocardium. Capillaries indicated with arrows. (e) MAC3 immunostained macrophage positive LV percentage area in WT (*n* = 4) and Chr4^D70/D70^ (*n* = 5) hearts at day 5 after MI. (f) Representative figures of MAC3 immunostained myocardium. Examples of positive, dark brown staining indicated with arrows. (g) CD4 immunostained T‐lymphocyte positive percentage area of LV in WT (*n* = 4) and Chr4^D70/D70^ (*n* = 5) hearts at day 5 after MI. (h) Representative figures of CD4 immunostained myocardium. Examples of positive, dark brow staining indicated with arrows. (i) Circulating white blood cell count (WBC) of Chr4^D70/D70^ and WT mice at day 5 (*n* = 4 + 3) and 4 weeks (*n* = 3 + 3) after MI. (j) Pathological condition of the Chr4^D70/D70^ and WT hearts at day 5 (*n* = 4 + 5) and after 4 weeks (*n* = 4 + 5) of MI, presented as heart instability index determined by dividing the instabilizing factors (MI area, inflammatory cell area, edema) with the stabilizing factors (capillary area, LV area). Statistical analysis for graphs (e) and (g) was performed by using *t*‐test, and for the rest of the graphs comparing more than two groups, two‐way ANOVA with Tukey's multiple comparisons test was used. Difference between groups was considered statistically significant when *p* < 0.05.

Comparison of the myocardial edema in acute and chronic MI revealed both Chr4^Δ70kb/Δ70kb^ (*p* = 0.008) and WT (*p* = 0.005) mice having significantly less edema in their LV myocardium after 4 weeks from the operation, in comparison to day 5 (Figure 4a,b). Capillary area, in turn, was significantly increased after 4 weeks in both genotypes (WT *p* = 0.005; Chr4^Δ70kb/Δ70^ *p* = 0.007) (Figure 4c,d). However, there were no significant differences in cardiac edema or LV revascularization between Chr4^Δ70kb/Δ70kb^ and WT mice, neither per se (Figure 4a–d) nor when normalized to infarction area (data not shown). As expected, inflammatory cells were detected only in acute MI, whereas at the 4 weeks' time point no positive staining for inflammatory cells was detected in the tissue (not shown). However, there was no difference in immune cell infiltration into the acute MI area (Figure 4e–h) nor in the circulating white blood cell count (Figure 4i) between the genotypes. Finally, to assess the severity of the pathology, a heart instability index was calculated by dividing the instabilizing characteristics (infarction area, inflammatory cell area and edema) with stabilizing factors (LV area, capillary area). In both Chr4^Δ70kb/Δ70kb^ (*p* = 0.0002) and WT (*p* > 0.001) mice, the instability index was significantly higher in acute MI and decreased in the more chronic 4 weeks' time point (Figure 4j).

### 3.3 Chr4^Δ70kb^ ^/Δ70kb^ does not affect the post‐MI cardiac function

**FIGURE 5:**
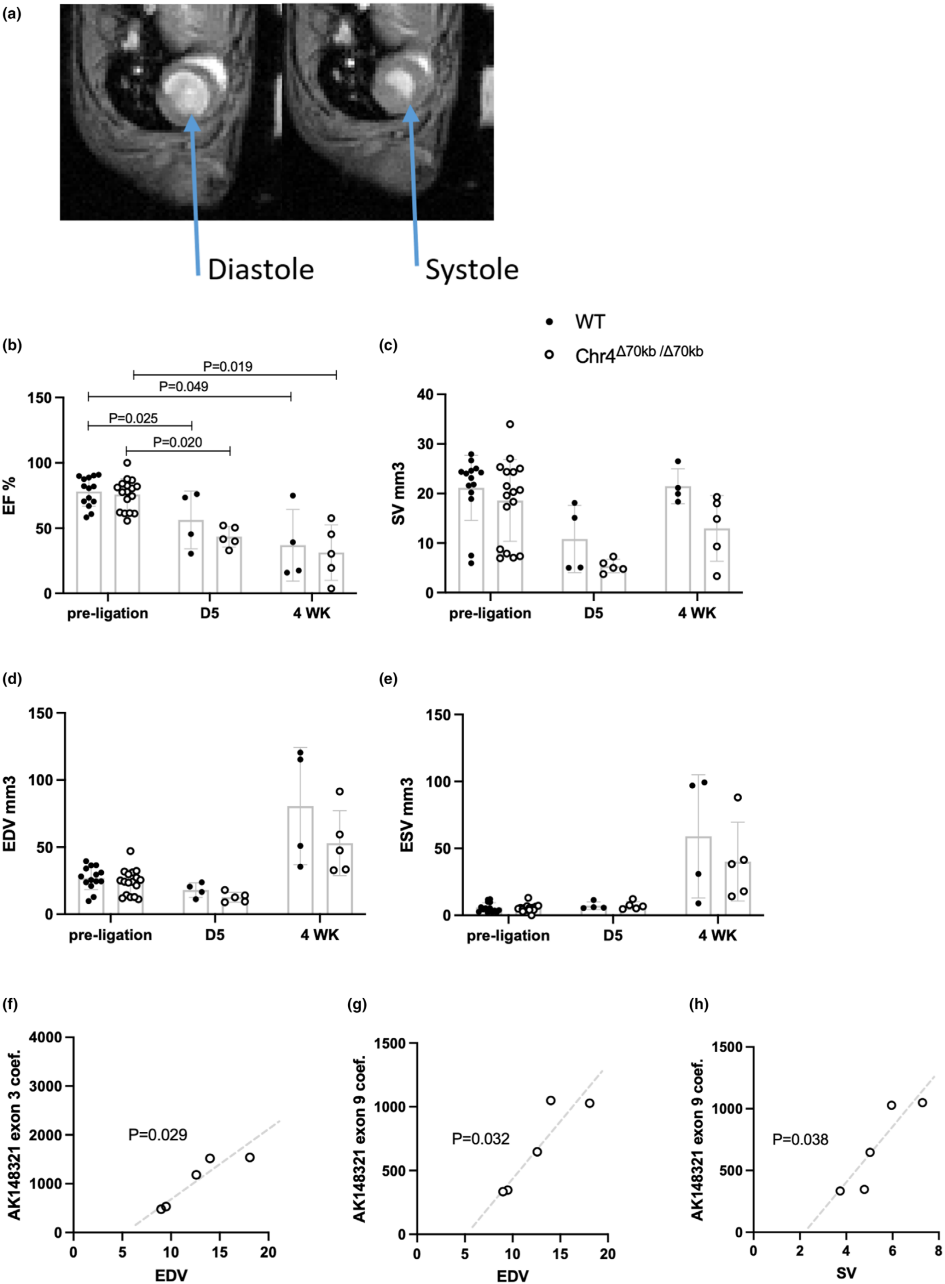
Cardiac function is impaired after myocardial infarction in mice. Expression of Ak148321 correlates with cardiac function in Chr4^d70/d70^ mice at acute MI. (a) Representative MRI of mouse cardiac cycle. (b) Ejection fraction (EF), (c) stroke volume (SV), (d) end diastolic volume (EDV), and (e) end systolic volume (ESV) of WT (*n* = 14) and Chr4^D70/D70^ (*n* = 17) mice before, after 5 days (*n* = 4 + 5), and after 4 weeks (*n* = 4 + 5) of MI. (f) Chr4^d70/d70^ (*n* = 5) relative cardiac mRNA expression of Ak148321 exon 3 and (g) exon 9 correlated with EDV, and (h) exon 9 correlated with SV at day 5 after MI. mRNA expression was measured with ddPCR, and data is normalized to an endogenous control *Rplp0*. Statistical analysis for cardiac function was performed by using two‐way mixed effects ANOVA with Tukey's multiple comparisons test. Statistical analysis for correlation was performed with Pearson *r* test. Differences were considered statistically significant when *p* < 0.05.

Diastolic and systolic function of the mouse hearts was determined both before and after MI with cardiac MRI (Figure 5a). As expected, 5 days after the MI, EF of the mice had significantly dropped in both WT (*p* = 0.025) and Chr4^Δ70kb/Δ70kb^ (*p* = 0.020) mice in comparison to their pre‐ligation values (Figure 5b). After 4 weeks of operation, EF of both WT (*p* = 0.049) and Chr4^Δ70kb/Δ70kb^ (*p* = 0.019) mice was still significantly lower in comparison to their EF preceding the MI. There were no statistically significant differences in SV of the hearts between the time points or animal groups (Figure 5c). In general, Chr4^Δ70kb/Δ70kb^ mice seemed to have lower SV than WT after 4 weeks of LAD ligation, but the difference did not reach statistical significance (*p* = 0.133). EDV and ESV of the LV were not significantly affected by MI (Figure 5d,e).

### 3.4 Cardiac expression of Ak148321 positively correlates with the cardiac function in Chr4^Δ70kb^ ^/Δ70kb^ mice after acute MI

To see whether the Ak148321 expression goes along with cardiac function, we ran a correlation analysis between the Ak148321 exons and the EF, EDV, ESV, and SV of the Chr4^Δ70kb/Δ70kb^ hearts. In Chr4^Δ70kb/Δ70kb^ mice with acute MI, the cardiac expression of the Ak148321 exons 3 (*p* = 0.029) and 9 (*p* = 0.032) was positively correlated with EDV (Figure 5f,g). Exon 9 was also positively correlated with SV in Chr4^Δ70kb/Δ70kb^ mice (*p* = 0.038) (Figure 5h). In chronic, 4‐week post‐MI time point, there were no significant correlations between the Ak148321 expression and cardiac function (data not shown).

## 4 DISCUSSION

9p21.3 CAD risk locus and its lncRNA transcript ANRIL have been identified as a hotspot for CAD and MI associated SNPs (Burd et al., 2010; Helgadottir et al., 2007; Holdt et al., 2016; McPherson et al., 2007; Samani et al., 2007; The Wellcome Trust Case Control Consortium, 2007; Tibaut et al., 2022). While at first both increased and decreased expression of ANRIL were reported within carriers of the risk haplotypes, it was quickly found that the risk might be modulated by the specific ANRIL isoforms, as its circular isoforms were associated with atheroprotection and linear isoforms with the promotion of atherogenesis (Burd et al., 2010). Recent clinical studies have provided further support to the atheroprotective role of circular ANRIL, but the conclusive understanding of ANRIL's expression patterns in different stages of CAD is still lacking (Razeghian‐Jahromi et al., 2022; Rodríguez‐Esparragón et al., 2023). In a 2023 study by Jiao et al., plasma levels of ANRIL were increased both in a stable *angina pectoris* and MI, and patients with stable *angina* showed even higher levels than patients with MI (Jiao et al., 2023). Overexpression of ANRIL has also been suggested to improve cardiac function by promoting postischemic angiogenesis (Huang, Pan, et al., 2020). In a preliminary study with 24 patients, a lower expression of ANRIL was associated with ventricular fibrillation, a common cause of sudden death, during acute MI (Pan‐Lizcano et al., 2021). However, these studies did not report the expression levels of the different ANRIL isoforms.

In the present study, we investigated the cardiac effects of deficiency in the 9p21.3 CAD risk locus murine orthologue in mice suffering from either acute or chronic MI. Survival rates of these Chr4^Δ70kb/Δ70kb^ mice after the surgically induced MI did not differ from the WT mice, which is in line with the data implying the 9p21.3 haplotype does not have an effect on mortality after a cardiac event (Gao et al., 2010; Patel et al., 2019). Both Chr4^Δ70kb/Δ70kb^ and WT mice showed cardiomyocyte loss, immune cell infiltration, and edema in their LV myocardium in the acute phase of MI, and the chronic condition led to the formation of fibrous scar and increased capillary area. In a meta‐analysis from 2019, 9p21.3 polymorphism was associated with the tissue revascularization after cardiac events (Patel et al., 2019). However, we did not see the risk locus deletion affecting the capillary area after MI in mice, and no effect in infarction size or cardiac function was detected either.

The closest protein‐coding genes neighboring the 9p21.3 CAD risk interval are the cell cycle regulators and tumor suppressors *MTAP*, *CDKN2A*, and *CDKN2B*, which play an important role in cell cycle regulation and inhibition of cell proliferation (Krimpenfort et al., 2007; Kryukov et al., 2016; Zhao et al., 2016). In humans, SNPs in *MTAP* and *CDKN2B* have been associated with MI, but their potential mechanisms remain unknown (Yang et al., 2009). Adult cardiomyocytes have very limited proliferation capacity; however, in cardiac injury, there is increased proliferation of other cell types, including endothelial cells, inflammatory cells, and fibroblasts (Shinde & Frangogiannis, 2014). When looking at chronic MI in mice, we saw no difference in the expression of *Cdkn2a*, Cdkn*2b*, or *Mtap* in the intact parts of the myocardium between genotypes. However, in the infarction scar area, consisting mostly of fibrous tissue, the expression of *Cdkn2a* was increased in WT mice, yet both *Cdkn2a* and *‐b* remained significantly lower in Chr4^Δ70kb/Δ70kb^ mice, suggesting that Ak148321 may regulate the *Cdkn2a/b* expression. Considering the proliferation‐suppressing role of the *Cdkn2a/b*, their downregulation in the MI scar of the Chr4^Δ70kb/Δ70kb^ mice could trigger angiogenesis and endothelial cell proliferation, but this would require additional studies and is not shown in the present study. In healthy Chr4^Δ70kb/Δ70kb^ mice, reduced cardiac expression of *Cdkn2/b* has been reported, but the expression of these genes seems to depend on cell type and environment, which is further supported by the present data, as the differences were visible only in the MI scar area (Kettunen et al., 2023, 2024; Visel et al., 2010).

In humans, the expression of linear ANRIL isoforms has been correlated with more severe cardiac disease outcomes, while the circular ANRIL has been associated with a protective phenotype, like a decrease in oxidative stress (Rodríguez‐Esparragón et al., 2023). Hence, we investigated the expression pattern of the murine ANRIL equivalent, Ak148321, in the infarcted myocardium and, as a reference, in a healthy WT mouse myocardium. In WT mice, we found a significantly higher expression of the Ak148321 exons and circular isoforms in infarcted myocardium, especially after the acute MI. This is likely caused by the high inflammatory cell infiltration to the MI area, because as previously reported, both human ANRIL and mouse Ak148321 are expressed especially in the inflammatory cells (Kettunen et al., 2023). Due to the deletion, Chr4^Δ70kb/Δ70kb^ mice do not express the middle exon of the Ak148321 nor the circular isoforms studied. However, the Chr4^Δ70kb/Δ70kb^ mice showed downregulation of the Ak148321 exons 3 and 9 in comparison to the WT mice in acute MI. Together, these results suggest that Ak148321 is playing a role in altering inflammatory cell gene function, as demonstrated previously (Kettunen et al., 2023). In the Chr4^Δ70kb/Δ70kb^ mice with acute MI, a correlation analysis revealed the Ak148321 exons 3 and 9 being associated with higher EDV and SV. In humans, increased ESV has been associated with heart failure (Kato et al., 2020). However, considering the relatively low number of replicates in both the gene expression analysis and post‐MI cardiac function imaging, more comprehensive research is needed to draw conclusions on the Ak148321 expression and the severity of MI. As there were no significant differences in cardiac function between the Chr4^Δ70kb/Δ70kb^ and WT mice, it is possible that the correlations detected are not causal, or more statistical power would be needed to reveal the potential effects of the genotype.

It has been demonstrated that the variability in coronary anatomy is commonly causes a variation in murine in vivo models for MI, which, along with the mentioned low number of replicates, forms a limitation for the present study (Chen et al., 2017). Moreover, in human a significant damage related to MI happens due to the re‐establishment of blood flow, causing a reperfusion injury, a phenomenon not demonstrated in the current mouse model with a permanent LAD‐ligation (Simonis et al., 2012). Nevertheless, our data suggest that the murine orthologous locus to human 9p21.3 is regulating the *Cdkn2a/b* expression in the chronically infarcted, fibrous mouse myocardium, and its long non‐coding RNA Ak148321 is being activated in an ischemic myocardial injury, which, along with the previous studies, provides further support for the role of Ak148321 in inflammatory cell activation.

The exact mechanisms of the risk locus activation and how it regulates gene expression have been disputed. In human lymphoblastoid and endothelial cell lines, the risk SNPs and ANRIL have been demonstrated to impair interferon‐γ signaling responses and function as components of the inflammatory NF‐κB pathway (Harismendy et al., 2011; Zhou et al., 2016). ANRIL has also been reported to regulate C*DKN2A/B* via direct interaction with Polycomb repressive complex 1 (*PRC1*) and 2 (*PRC2*), which are known epigenetic regulators responding to various cellular and environmental signals, like hypoxia (Harada et al., 2024; Kotake et al., 2011; Mahara et al., 2016; Yap et al., 2010). In mice, data is even more scarce. Thus, more extensive research is needed to identify the regulatory pathways related to ANRIL/Ak148321 in both healthy and ischemic myocardium. Nevertheless, in the present study, the deficiency in the risk locus did not significantly affect cardiac function, survival, nor recovery of the mice after MI, aligning with the idea of the locus and its lncRNA transcripts regulating mostly atherogenesis predisposing to cardiac events like MI, rather than MI itself (Horne et al., 2008; Patel et al., 2014, 2019).

## FUNDING INFORMATION

This research was supported by Research Council of Finland GeneCellNano Flagship Program (S‐YH), Onni ja Hilja Tuovinen foundation (S.K.), The Finnish Foundation for Cardiovascular Research (A.‐K.R. and E.Y.‐H.), Orion Research Foundation (S.K. and A.‐K.R.), Yrjö Jahnsson Foundation (S.K.), Emil Aaltonen Foundation (T.S. and E.Y.‐H.), Finnish Cultural Foundation (E.Y.‐H.), State Research Funding (E.Y.‐H.), and Research Council of Finland (A.K.R.).

## ETHICS STATEMENT

Animal experiments were approved by the National Experimental Animal Board of Finland and carried out following the guidelines of the Finnish Act on Animal Experimentation and Directive 2010/63/EU of the European Parliament.

## Supporting information


Tables S1–S2.

## Data Availability

All data are presented in the article text, figures, and tables. Raw data are available upon a reasonable request from the corresponding author.

## References

[phy270344-bib-0001] Burd, C. E. , Jeck, W. R. , Liu, Y. , Sanoff, H. K. , Wang, Z. , & Sharpless, N. E. (2010). Expression of linear and novel circular forms of an INK4/ARF‐associated non‐coding RNA correlates with atherosclerosis risk. PLoS Genetics, 6, e1001233. 10.1371/journal.pgen.1001233 21151960 PMC2996334

[phy270344-bib-0002] Chen, J. , Ceholski, D. K. , Liang, L. , Fish, K. , & Hajjar, R. J. (2017). Variability in coronary artery anatomy affects consistency of cardiac damage after myocardial infarction in mice. American Journal of Physiology. Heart and Circulatory Physiology, 313, H275–H282. 10.1152/ajpheart.00127.2017 28550174 PMC5582916

[phy270344-bib-0003] Cheng, J. , Cai, M.‐Y. , Chen, Y.‐N. , Li, Z.‐C. , Tang, S.‐S. , Yang, X.‐L. , Chen, C. , Liu, X. , & Xiong, X. D. (2017). Variants in ANRIL gene correlated with its expression contribute to myocardial infarction risk. Oncotarget, 8(8), 12607–12619. 10.18632/oncotarget.14721 28107200 PMC5355039

[phy270344-bib-0004] Cho, H. , Shen, G.‐Q. , Wang, X. , Wang, F. , Archacki, S. , Li, Y. , Yu, G. , Chakrabarti, S. , Chen, Q. , & Wang, Q. K. (2019). Long noncoding RNA ANRIL regulates endothelial cell activities associated with coronary artery disease by up‐regulating CLIP1, EZR, and LYVE1 genes. The Journal of Biological Chemistry, 294, 3881–3898. 10.1074/jbc.RA118.005050 30655286 PMC6422082

[phy270344-bib-0005] Christ, C. , Ocskay, Z. , Kovács, G. , & Jakus, Z. (2024). Characterization of atherosclerotic mice reveals a sex‐dependent susceptibility to plaque calcification but no major changes in the lymphatics in the arterial wall. International Journal of Molecular Sciences, 25, 4046. 10.3390/ijms25074046 38612867 PMC11012298

[phy270344-bib-0006] Gao, E. , Lei, Y. H. , Shang, X. , Huang, Z. M. , Zuo, L. , Boucher, M. , Fan, Q. , Chuprun, J. K. , Ma, X. L. , & Koch, W. J. (2010). A novel and efficient model of coronary artery ligation and myocardial infarction in the mouse. Circulation Research, 107, 1445–1453. 10.1161/CIRCRESAHA.110.223925 20966393 PMC3005817

[phy270344-bib-0007] Harada, M. , Su‐Harada, K. , Kimura, T. , Ono, K. , & Ashida, N. (2024). Sustained activation of NF‐κB through constitutively active IKKβ leads to senescence bypass in murine dermal fibroblasts. Cell Cycle, 23, 308–327. 10.1080/15384101.2024.2325802 38461418 PMC11057680

[phy270344-bib-0008] Harismendy, O. , Notani, D. , Song, X. , Rahim, N. G. , Tanasa, B. , Heintzman, N. , Ren, B. , Fu, X. D. , Topol, E. J. , Rosenfeld, M. G. , & Frazer, K. A. (2011). 9p21 DNA variants associated with coronary artery disease impair interferon‐γ signalling response. Nature, 470, 264–268. 10.1038/nature09753 21307941 PMC3079517

[phy270344-bib-0009] Haslacher, H. , Perkmann, T. , Ratzinger, F. , Grimm, G. , Exner, M. , Keller, A. , Schmetterer, K. , Priemer, C. , Endler, G. , Wagner, O. , & Schillinger, M. (2016). 9p21.3 risk locus is associated with first‐ever myocardial infarction in an Austrian cohort. Journal of Cardiovascular Medicine, 17, 595–600. 10.2459/JCM.0000000000000183 25032714

[phy270344-bib-0010] Helgadottir, A. , Thorleifsson, G. , Manolescu, A. , Gretarsdottir, S. , Blondal, T. , Jonasdottir, A. , Jonasdottir, A. , Sigurdsson, A. , Baker, A. , Palsson, A. , Masson, G. , Gudbjartsson, D. F. , Magnusson, K. P. , Andersen, K. , Levey, A. I. , Backman, V. M. , Matthiasdottir, S. , Jonsdottir, T. , Palsson, S. , … Stefansson, K. (2007). A common variant on chromosome 9p21 affects the risk of myocardial infarction. Science, 316, 1491–1493. 10.1126/science.1142842 17478679

[phy270344-bib-0011] Holdt, L. M. , Sass, K. , Gäbel, G. , Bergert, H. , Thiery, J. , & Teupser, D. (2011). Expression of Chr9p21 genes CDKN2B (p15INK4b), CDKN2A (p16INK4a, p14ARF) and MTAP in human atherosclerotic plaque. Atherosclerosis, 214, 264–270. 10.1016/j.atherosclerosis.2010.06.029 20637465

[phy270344-bib-0012] Holdt, L. M. , Stahringer, A. , Sass, K. , Pichler, G. , Kulak, N. A. , Wilfert, W. , Kohlmaier, A. , Herbst, A. , Northoff, B. H. , Nicolaou, A. , Gäbel, G. , Beutner, F. , Scholz, M. , Thiery, J. , Musunuru, K. , Krohn, K. , Mann, M. , & Teupser, D. (2016). Circular non‐coding RNA ANRIL modulates ribosomal RNA maturation and atherosclerosis in humans. Nature Communications, 7, 12429. 10.1038/ncomms12429 PMC4992165 27539542

[phy270344-bib-0013] Horne, B. D. , Carlquist, J. F. , Muhlestein, J. B. , Bair, T. L. , & Anderson, J. L. (2008). Association of Variation in the chromosome 9p21 locus with myocardial infarction versus chronic coronary artery disease. Circulation. Cardiovascular Genetics, 1, 85–92. 10.1161/CIRCGENETICS.108.793158 19956784 PMC2745117

[phy270344-bib-0014] Huang, Q. , Pan, M. , Zhou, J. , & Yin, F. (2020). Overexpression of long non‐coding RNA ANRIL promotes post‐ischaemic angiogenesis and improves cardiac functions by targeting Akt. Journal of Cellular and Molecular Medicine, 24, 6860–6868. 10.1111/jcmm.15343 32400082 PMC7299705

[phy270344-bib-0015] Huang, T. , Zhao, H.‐Y. , Zhang, X.‐B. , Gao, X.‐L. , Peng, W.‐P. , Zhou, Y. , Zhao, W. H. , & Yang, H. F. (2020). LncRNA ANRIL regulates cell proliferation and migration via sponging miR‐339‐5p and regulating FRS2 expression in atherosclerosis. European Review for Medical and Pharmacological Sciences, 24(4), 1956–1969. 10.26355/eurrev_202002_20373 32141564

[phy270344-bib-0016] Jiao, Y. , Meng, F. , Ma, G. , Lei, H. , & Liu, J. (2023). An increase in a long noncoding RNA ANRIL in peripheral plasma is an indicator of stable angina. Clinics, 78, 100289. 10.1016/j.clinsp.2023.100289 37852142 PMC10585623

[phy270344-bib-0017] Kato, M. , Kitada, S. , Kawada, Y. , Nakasuka, K. , Kikuchi, S. , Seo, Y. , & Ohte, N. (2020). Left ventricular end‐systolic volume is a reliable predictor of new‐onset heart failure with preserved left ventricular ejection fraction. Cardiology Research and Practice, 2020, 3106012. 10.1155/2020/3106012 32670635 PMC7341373

[phy270344-bib-0018] Kettunen, S. , Ruotsalainen, A.‐K. , Örd, T. , Suoranta, T. , Heikkilä, J. , Kaikkonen, M. U. , Laham‐Karam, N. , & Ylä‐Herttuala, S. (2023). Deletion of the murine ortholog of human 9p21.3 locus promotes atherosclerosis by increasing macrophage proinflammatory activity. Frontiers in Cardiovascular Medicine, 10, 1113890. 10.3389/fcvm.2023.1113890 36950286 PMC10025322

[phy270344-bib-0019] Kettunen, S. , Suoranta, T. , Beikverdi, S. , Heikkilä, M. , Slita, A. , Räty, I. , Ylä‐Herttuala, E. , Öörni, K. , Ruotsalainen, A. K. , & Ylä‐Herttuala, S. (2024). Deletion of the murine ortholog of the human 9p21.3 locus leads to insulin resistance and obesity in hypercholesterolemic mice. Cells, 13, 983. 10.3390/cells13110983 38891115 PMC11171903

[phy270344-bib-0020] Kojima, Y. , Ye, J. , Nanda, V. , Wang, Y. , Flores, A. M. , Jarr, K.‐U. , Tsantilas, P. , Guo, L. , Finn, A. V. , Virmani, R. , & Leeper, N. J. (2020). Knockout of the murine ortholog to the human 9p21 coronary artery disease locus leads to smooth muscle cell proliferation, vascular calcification, and advanced atherosclerosis. Circulation, 141, 1274–1276. 10.1161/CIRCULATIONAHA.119.043413 32282248 PMC7158761

[phy270344-bib-0021] Kotake, Y. , Nakagawa, T. , Kitagawa, K. , Suzuki, S. , Liu, N. , Kitagawa, M. , & Xiong, Y. (2011). Long non‐coding RNA ANRIL is required for the PRC2 recruitment to and silencing of p15INK4B tumor suppressor gene. Oncogene, 30, 1956–1962. 10.1038/onc.2010.568 21151178 PMC3230933

[phy270344-bib-0022] Krimpenfort, P. , IJpenberg, A. , Song, J.‐Y. , Van Der Valk, M. , Nawijn, M. , Zevenhoven, J. , & Berns, A. (2007). p15Ink4b is a critical tumour suppressor in the absence of p16Ink4a. Nature, 448(7156), 943–946. 10.1038/nature06084 17713536

[phy270344-bib-0023] Kryukov, G. V. , Wilson, F. H. , Ruth, J. R. , Paulk, J. , Tsherniak, A. , Marlow, S. E. , Vazquez, F. , Weir, B. A. , Fitzgerald, M. E. , Tanaka, M. , Bielski, C. M. , Scott, J. M. , Dennis, C. , Cowley, G. S. , Boehm, J. S. , Root, D. E. , Golub, T. R. , Clish, C. B. , Bradner, J. E. , … Garraway, L. A. (2016). MTAP deletion confers enhanced dependency on the PRMT5 arginine methyltransferase in cancer cells. Science, 351, 1214–1218. 10.1126/science.aad5214 26912360 PMC4997612

[phy270344-bib-0024] Lusis, A. J. (2000). Atherosclerosis. Nature, 407, 233–241. 10.1038/35025203 11001066 PMC2826222

[phy270344-bib-0025] Mahara, S. , Lee, P. L. , Feng, M. , Tergaonkar, V. , Chng, W. J. , & Yu, Q. (2016). HIFI‐α activation underlies a functional switch in the paradoxical role of Ezh2/PRC2 in breast cancer. Proceedings of the National Academy of Sciences of the United States of America, 113(26), E3735–E3744. 10.1073/pnas.1602079113 27303043 PMC4932959

[phy270344-bib-0026] McPherson, R. , Pertsemlidis, A. , Kavaslar, N. , Stewart, A. , Roberts, R. , Cox, D. R. , Hinds, D. A. , Pennacchio, L. A. , Tybjaerg‐Hansen, A. , Folsom, A. R. , Boerwinkle, E. , Hobbs, H. H. , & Cohen, J. C. (2007). A common allele on chromosome 9 associated with coronary heart disease. Science, 316, 1488–1491. 10.1126/science.1142447 17478681 PMC2711874

[phy270344-bib-0027] Pan‐Lizcano, R. , Nunez, L. , Pinon, P. , Flores, X. , Aldama, G. , Calvino, R. , Vazquez‐Rodriguez, J. M. , & Hermida, M. (2021). lncRNA CDKN2B‐AS1 associated to ventricular fibrillation during acute myocardial infarction. European Heart Journal, 42(Supplement_1), ehab724.0623. 10.1093/eurheartj/ehab724.0623

[phy270344-bib-0028] Patel, R. S. , Asselbergs, F. W. , Quyyumi, A. A. , Palmer, T. M. , Finan, C. I. , Tragante, V. , Deanfield, J. , Hemingway, H. , Hingorani, A. D. , & Holmes, M. V. (2014). Genetic variants at chromosome 9p21 and risk of first versus subsequent coronary heart disease events. Journal of the American College of Cardiology, 63, 2234–2245. 10.1016/j.jacc.2014.01.065 24607648 PMC4035794

[phy270344-bib-0029] Patel, R. S. , Schmidt, A. F. , Tragante, V. , McCubrey, R. O. , Holmes, M. V. , Howe, L. J. , Direk, K. , Åkerblom, A. , Leander, K. , Virani, S. S. , & Kaminski, K. A. (2019). Association of chromosome 9p21 with subsequent coronary heart disease events: A GENIUS‐CHD study of individual participant data. Circulation: Genomic and Precision Medicine, 12, e002471. 10.1161/CIRCGEN.119.002471 30897348 PMC6625876

[phy270344-bib-0030] Razeghian‐Jahromi, I. , Zibaeenezhad, M. J. , Karimi Akhormeh, A. , & Dara, M. (2022). Expression ratio of circular to linear ANRIL in hypertensive patients with coronary artery disease. Scientific Reports, 12, 1802. 10.1038/s41598-022-05731-9 35110626 PMC8810852

[phy270344-bib-0031] Robinet, P. , Milewicz, D. M. , Cassis, L. A. , Leeper, N. J. , Lu, H. S. , & Smith, J. D. (2018). Consideration of sex differences in design and reporting of experimental arterial pathology studies—Statement from ATVB council. Arteriosclerosis, Thrombosis, and Vascular Biology, 38, 292–303. 10.1161/ATVBAHA.117.309524 29301789 PMC5785439

[phy270344-bib-0032] Rodríguez‐Esparragón, F. , Torres‐Mata, L. B. , Cazorla‐Rivero, S. E. , Serna Gómez, J. A. , González Martín, J. M. , Cánovas‐Molina, Á. , Medina‐Suárez, J. A. , González‐Hernández, A. N. , Estupiñán‐Quintana, L. , Bartolomé‐Durán, M. C. , Rodríguez‐Pérez, J. C. , & Varas, B. C. (2023). Analysis of ANRIL isoforms and key genes in patients with severe coronary artery disease. International Journal of Molecular Sciences, 24, 16127. 10.3390/ijms242216127 38003316 PMC10671206

[phy270344-bib-0033] Samani, N. J. , Erdmann, J. , Hall, A. S. , Hengstenberg, C. , Mangino, M. , Mayer, B. , Dixon, R. J. , Meitinger, T. , Braund, P. , Wichmann, H. E. , Barrett, J. H. , König, I. R. , Stevens, S. E. , Szymczak, S. , Tregouet, D. A. , Iles, M. M. , Pahlke, F. , Pollard, H. , Lieb, W. , … WTCCC and the Cardiogenics Consortium . (2007). Genomewide association analysis of coronary artery disease. The New England Journal of Medicine, 357, 443–453. 10.1056/NEJMoa072366 17634449 PMC2719290

[phy270344-bib-0034] Schindelin, J. , Arganda‐Carreras, I. , Frise, E. , Kaynig, V. , Longair, M. , Pietzsch, T. , Preibisch, S. , Rueden, C. , Saalfeld, S. , Schmid, B. , Tinevez, J. Y. , White, D. J. , Hartenstein, V. , Eliceiri, K. , Tomancak, P. , & Cardona, A. (2012). Fiji: An open‐source platform for biological‐image analysis. Nature Methods, 9, 676–682. 10.1038/nmeth.2019 22743772 PMC3855844

[phy270344-bib-0035] Shinde, A. V. , & Frangogiannis, N. G. (2014). Fibroblasts in myocardial infarction: A role in inflammation and repair. Journal of Molecular and Cellular Cardiology, 70, 74–82. 10.1016/j.yjmcc.2013.11.015 24321195 PMC3995820

[phy270344-bib-0036] Simonis, G. , Strasser, R. H. , & Ebner, B. (2012). Reperfusion injury in acute myocardial infarction. Critical Care (London, England), 16(S2), A22. 10.1186/cc11280

[phy270344-bib-0037] The Wellcome Trust Case Control Consortium . (2007). Genome‐wide association study of 14,000 cases of seven common diseases and 3,000 shared controls. Nature, 447, 661–678. 10.1038/nature05911 17554300 PMC2719288

[phy270344-bib-0038] Tibaut, M. , Naji, F. , & Petrovič, D. (2022). Association of myocardial infarction with CDKN2B antisense RNA 1 (CDKN2B‐AS1) rs1333049 polymorphism in slovenian subjects with type 2 diabetes mellitus. Genes, 13, 526. 10.3390/genes13030526 35328079 PMC8952457

[phy270344-bib-0039] Vaduganathan, M. , Mensah, G. A. , Turco, J. V. , Fuster, V. , & Roth, G. A. (2022). The global burden of cardiovascular diseases and risk. Journal of the American College of Cardiology, 80, 2361–2371. 10.1016/j.jacc.2022.11.005 36368511

[phy270344-bib-0040] Vausort, M. , Wagner, D. R. , & Devaux, Y. (2014). Long noncoding RNAs in patients with acute myocardial infarction. Circulation Research, 115, 668–677. 10.1161/CIRCRESAHA.115.303836 25035150

[phy270344-bib-0041] Visel, A. , Zhu, Y. , May, D. , Afzal, V. , Gong, E. , Attanasio, C. , Blow, M. J. , Cohen, J. C. , Rubin, E. M. , & Pennacchio, L. A. (2010). Targeted deletion of the 9p21 non‐coding coronary artery disease risk interval in mice. Nature, 464, 409–412. 10.1038/nature08801 20173736 PMC2938076

[phy270344-bib-0042] Wahlstrand, B. , Orho‐Melander, M. , Delling, L. , Kjeldsen, S. , Narkiewicz, K. , Almgren, P. , Hedner, T. , & Melander, O. (2009). The myocardial infarction associated CDKN2A/CDKN2B locus on chromosome 9p21 is associated with stroke independently of coronary events in patients with hypertension. Journal of Hypertension, 27, 769–773. 10.1097/HJH.0b013e328326f7eb 19293724

[phy270344-bib-0043] Yang, J. , Huang, X. , Hu, F. , Fu, X. , Jiang, Z. , & Chen, K. (2019). LncRNA ANRIL knockdown relieves myocardial cell apoptosis in acute myocardial infarction by regulating IL‐33/ST2. Cell Cycle, 18, 3393–3403. 10.1080/15384101.2019.1678965 31674275 PMC6927712

[phy270344-bib-0044] Yang, X.‐C. , Zhang, Q. , Chen, M.‐L. , Li, Q. , Yang, Z.‐S. , Li, L. , Cao, F. F. , Chen, X. D. , Liu, W. J. , Jin, L. , & Wang, X. F. (2009). MTAP and CDKN2B genes are associated with myocardial infarction in Chinese Hans. Clinical Biochemistry, 42, 1071–1075. 10.1016/j.clinbiochem.2009.02.021 19272367

[phy270344-bib-0045] Yap, K. L. , Li, S. , Muñoz‐Cabello, A. M. , Raguz, S. , Zeng, L. , Mujtaba, S. , Gil, J. , Walsh, M. J. , & Zhou, M. M. (2010). Molecular interplay of the noncoding RNA ANRIL and methylated histone H3 lysine 27 by polycomb CBX7 in transcriptional silencing of INK4a. Molecular Cell, 38, 662–674. 10.1016/j.molcel.2010.03.021 20541999 PMC2886305

[phy270344-bib-0046] Ylä‐Herttuala, S. , Palinski, W. , Rosenfeld, M. E. , Parthasarathy, S. , Carew, T. E. , Butler, S. , Witztum, J. L. , & Steinberg, D. (1989). Evidence for the presence of oxidatively modified low density lipoprotein in atherosclerotic lesions of rabbit and man. The Journal of Clinical Investigation, 84, 1086–1095. 10.1172/JCI114271 2794046 PMC329764

[phy270344-bib-0047] Zeng, R. , Song, X.‐J. , Liu, C.‐W. , & Ye, W. (2019). LncRNA ANRIL promotes angiogenesis and thrombosis by modulating microRNA‐99a and microRNA‐449a in the autophagy pathway. American Journal of Translational Research, 11, 7441–7448. 31934291 PMC6943445

[phy270344-bib-0048] Zhao, R. , Choi, B. Y. , Lee, M.‐H. , Bode, A. M. , & Dong, Z. (2016). Implications of genetic and epigenetic alterations of CDKN2A (p16 INK4a) in cancer. eBioMedicine, 8, 30–39. 10.1016/j.ebiom.2016.04.017 27428416 PMC4919535

[phy270344-bib-0049] Zhou, X. , Han, X. , Wittfeldt, A. , Sun, J. , Liu, C. , Wang, X. , Gan, L. M. , Cao, H. , & Liang, Z. (2016). Long non‐coding RNA ANRIL regulates inflammatory responses as a novel component of NF‐κB pathway. RNA Biology, 13, 98–108. 10.1080/15476286.2015.1122164 26618242 PMC4829310

[phy270344-bib-0050] Zivotić, I. , Djurić, T. , Stanković, A. , Milasinovic, D. , Stankovic, G. , Dekleva, M. , Marković Nikolić, N. , Alavantić, D. , & Zivković, M. (2019). CDKN2B gene expression is affected by 9p21.3 rs10757278 in CAD patients, six months after the MI. Clinical Biochemistry, 73, 70–76. 10.1016/j.clinbiochem.2019.08.003 31386834

