# Supplementary material for: Myocardial infarction activates the 9p21.3 orthologous locus expression, but its absence does not alter cardiac pathophysiology in ischemia
Source: Physiol Rep. 2025 May 22;13(10):e70344. doi: 10.14814/phy2.70344 (PMC12098971; doi:10.14814/phy2.70344)
Supplement: Supplementary file 1 — Tables S1–S2. [file PHY2-13-e70344-s001.docx]

**SUPPLEMENTAL MATERIAL**

**Supplemental table 1. *MRI imaging parameters.***

| **Magnet strength** | **7 T** | **9.4 T** |
| --- | --- | --- |
| **Maximum strength of gradient set** | 760 mT/m | 600 mT/m |
| **Sequence** | FLASH | FLASH |
| **Coil** | surface | volume |
| **Field of view** | 25 mm x 25 mm | 30 mm x 30 mm  40 mm x 40 mm |
| **Slice thickness** | 1 mm | 1 mm |
| **Matrix size** | 192 x 192  256 x 256 | 192 x 192  128 x 128 |
| **Echo time (TE)** | 1.6 - 3 ms | 1.9 - 3 ms |
| **Repetition time (TR)** | 7-8 ms | 4,5 ms |
| **Flip angle** | 10- 15 ^o^ | 10- 20 ^o^ |

**Supplemental table 2. *Pre-designed and custom qPCR assays used for measuring gene expression.***

| **Species** | **Target** | **Manufacturer** | **Product code** | |
| --- | --- | --- | --- | --- |
| *Mus musculus* | *Cdkn2a* | Integrated DNA Technologies | Mm.PT.58.43961185 | |
| *Mus musculus* | *Cdkn2b* | Integrated DNA Technologies | Mm.PT.58.7138437 | |
| *Mus musculus* | *Hprt1* | Integrated DNA Technologies | Mm.PT.39a.22214828 | |
| *Mus musculus* | *Mtap* | Integrated DNA Technologies | Mm.PT.56a.29238418 | |
| *Mus musculus* | *Rplp0* | Integrated DNA Technologies | Mm.PT.58.43894205 | |
|  |  |  |  |  |
| **Species** | **Target** | **Manufacturer** |  | **Sequence** |
| *Mus musculus* | Ak148321 Exon3 | Integrated DNA Technologies | Primer 1 | GCA CCT GGG TAG ATG TTC TTT |
|  |  |  | Primer 2 | GTG TGG TCT TCG TAG CAG AAA |
|  |  |  | Probe | AG CTT CTC AG AGC CAA ACC GTC AT |
| *Mus musculus* | Ak148321 Exon6 | Integrated DNA Technologies | Primer 1 | GGC TAT ATA CTC ACC TCG GAA GA |
|  |  |  | Primer 2 | GAG GTA AAC CAG ATG CAG AAA GA |
|  |  |  | Probe | AA CCC TGC AT GTT CTC TCT CAG CC |
| *Mus musculus* | Ak148321 Exon9 | Integrated DNA Technologies | Primer 1 | CCC TGG CAC ATC ATA AGC TAT T |
|  |  |  | Primer 2 | GCC ATA CAG TGT CTT CTC TTC C |
|  |  |  | Probe | TG AGT CAT CG GAA TTC TGT GGT TTG CA |
| *Mus musculus* | Ak148321 Circular 1 | Integrated DNA Technologies | Primer 1 | GAC TAT CTC ACC ACT GGG ATT C |
|  | (chr4:89047023-89066563) |  | Primer 2 | TCT TCT TGC AAG TCC ATC CC |
|  |  |  | Probe | TGA CCT GAA TCT AAC TCC TTT GTT CAG CC |
| *Mus musculus* | Ak148321 Circular 2 | Integrated DNA Technologies | Primer 1 | GAC TAT CTC ACC ACT GGG ATT C |
|  | (chr4:89049887-89066563) |  | Primer 2 | CCA GCC TTG GCT TTG TTA AG |
|  |  |  | Probe | AGG CTG AAC AAA GGA GTT AGA TTC AGG T |
